# Central Venous Catheter-directed Tissue Plasminogen Activator in Massive Pulmonary Embolism

**DOI:** 10.5811/cpcem.2017.11.35845

**Published:** 2018-01-18

**Authors:** Vishal Gulati, Jared Brazg

**Affiliations:** Maimonides Medical Center, Department of Emergency Medicine, Brooklyn, New York

## Abstract

We present the case of an 88-year-old female who presented to the emergency department (ED) with suspected massive pulmonary embolism (PE) causing respiratory failure, right heart strain, and shock, who despite early and aggressive resuscitation with vasopressors and continuous peripheral infusion of tissue plasminogen activator (tPA), suffered a cardiac arrest in the ED. We describe the approach of a tPA bolus directed through a central venous catheter, resulting in return of spontaneous circulation and immediate improvement in physiologic parameters prior to confirmation of PE with computed tomography angiogram. We further hypothesize that in patients deemed too unstable to be transferred for embolectomy or catheter-directed thrombolysis, central venous catheter-directed bolus tPA may be more effective than peripheral infusion alone.

## INTRODUCTION

Pulmonary embolism (PE) is a common life-threatening cardiovascular condition encountered by emergency physicians, with presentations ranging from incidental asymptomatic sub-segmental pulmonary embolus to massive saddle embolus and circulatory collapse. In general, thrombolysis and surgical thrombectomy are reserved for cases of massive PE, as defined by acute PE associated with persistent hypotension or shock. Thrombolytic regimens, however, vary among different studies with no clear recommendation on ideal dosage or route of administration. The American College of Emergency Physicians (ACEP) clinical policy on management of PE gives a level B recommendation to “administer thrombolytic therapy in hemodynamically unstable patients with confirmed PE for whom the benefits of treatment outweigh the risks of life-threatening bleeding complications,” and a level C recommendation to “consider thrombolytic therapy in hemodynamically unstable patients with a high clinical suspicion for PE for whom the diagnosis of PE cannot be confirmed in a timely manner.”^1^ The following case demonstrates the successful administration of tissue plasminogen activator (tPA) through a central venous catheter (CVC) in an elderly female with massive PE.

## CASE REPORT

**Image 1:**
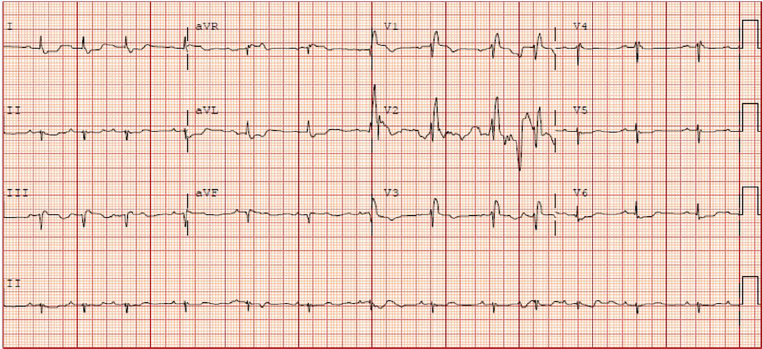
Initial 12-lead electrocardiogram showing signs of right heart strain including right bundle branch block and right axis deviation in patient found to have large pulmonary embolism.

The patient was an 88-year-old female with medical history significant for hypertension and hyperlipidemia who presented to the emergency department (ED) following a respiratory arrest. The patient was at home and had a syncopal episode while getting dressed. Paramedics arrived to find the patient hypoxic and in respiratory failure. She was intubated after receiving 10 milligrams of diazepam intravenously and transported to our ED. Her pre-hospital capillary glucose measurement was 230 milligrams per deciliter (mg/dL). Her presenting exam was notable for an obese elderly female, intubated and sedated with heart rate of 92 beats per minute (bpm), blood pressure 53/40 millimeters of mercury (mmHg), respiratory rate 21 breaths per minute by manual bag, end-tidal carbon dioxide (CO_2_) 14, and oxygen saturation of 88% on 100% fraction of inspired oxygen (FiO_2_). Her pupils were equal and reactive, and her heart, lung, and abdominal exam was unremarkable. She had cool extremities with peripheral cyanosis but palpable distal pulses. No spontaneous extremity movements were noted. A 12-lead electrocardiogram (ECG) showed a right bundle branch block without signs of acute myocardial infarction and no prior ECG for comparison (Image 1).

**Image 2:**
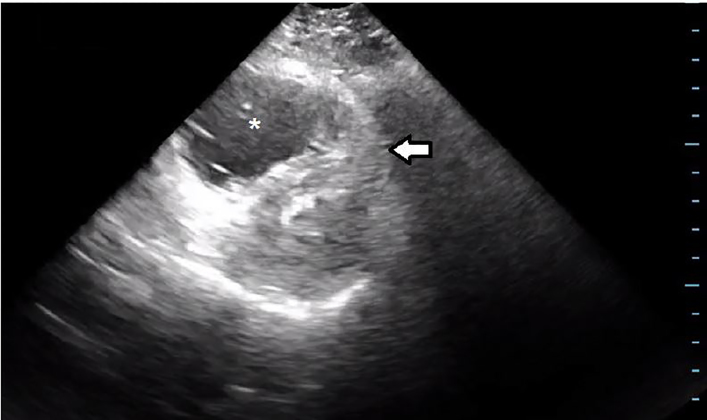
Point-of-care cardiac ultrasound, short-axis view demonstrating right ventricular dilation (asterisk) and paradoxical septal position (arrow).

In addition to a fluid bolus, a peripheral norepinephrine infusion and 1 milligram per minute of 50 milligrams tPA was started as an intravenous (IV) piggy-back while a CVC was being prepared. A bedside echocardiogram showed significant dilation of the right ventricle (Image 2).

**Image 3:**
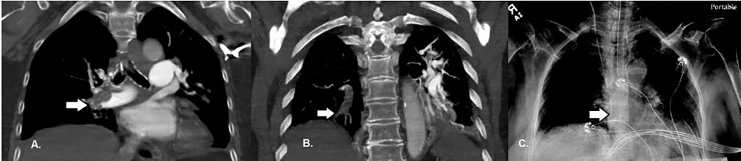
Coronal computed tomographic angiography in coronal view with arrows demonstrating A) large right main pulmonary artery, and B) subsegmental pulmonary embolisms; and C) portable chest radiograph showing catheter tip position in the superior vena cava (arrow).

Given the sudden cardiac arrest with hypoxia, persistent hypotension, and the echocardiogram findings with no acute myocardial infarction on the ECG, the most likely diagnosis was thought to be massive PE. The patient did not have any significant response to the initial infusion of thrombolytics. A right internal jugular CVC was inserted under sterile conditions. Moments later, the patient became bradycardic and then pulseless. Cardiopulmonary resuscitation (CPR) was initiated and two minutes of chest compressions were performed to complete the first round. Subsequently, the decision was made to push a 50mg tPA bolus through the CVC. CPR continued for four additional minutes and the patient had a total of 1 milligram of epinephrine and 75 milliequivalents of bicarbonate given, after which return of spontaneous circulation (ROSC) was achieved. Almost immediately following ROSC, the patient’s hemodynamics improved to a heart rate 87bpm, respiratory rate of 20, blood pressure 139/67mmHg, and oxygen saturation 99% on the ventilator. She was also following commands and moving all extremities. A computed tomography angiogram (CTA) of the chest showed extensive acute thromboembolic clot burden involving the right and left main, lobar, segmental, and subsegmental arteries (Image 3).

CPC-EM Capsule

What do we already know about this clinical entity?

Death from massive pulmonary embolism (PE) is due to hemodynamic collapse. The only emergency department intervention that likely improves survival rates in massive PE is thrombolysis.

What makes this presentation of disease reportable?

Not much is known about the ideal route and dose of tissue plasminogen activator for patients with massive PE who have hemodynamic collapse.

What is the major learning point?

We suggest a central catheter-directed thrombolytic bolus to improve drug efficacy in patients with cardiac arrest from massive PE.

How might this improve emergency medicine practice?

This specific treatment modality may reduce mortality rates in patients with massive PE.

The patient was admitted to the medical intensive care unit for further management. She was discharged home on hospital day 13 at her baseline functional status on oral apixaban.

## DISCUSSION

We present a case of massive PE that was strongly suspected based on history and physical exam and supported by the use of point-of-care ultrasound. The resulting hemodynamic collapse in this elderly female was thought to be successfully treated with CVC-directed tPA. The patient had return of cardiac function, resolution of hypoxia, and ultimately had a favorable outcome. It has been described that patients with massive PE and right ventricular dysfunction have a more rapid return of ventricular function and pulmonary perfusion with a thrombolytic bolus.^2^ Guidelines from the American Heart Association support that fibrinolysis is reasonable in patients with suspected massive PE, and is associated with an acceptable risk of bleeding complications.^3^ This strategy has also been endorsed by the American College of Chest Physicians and ACEP.^1^, ^4^

For patients with massive PE and hemodynamic instability, poor peripheral perfusion from right sided heart failure may limit tPA efficacy if administered through a peripheral intravenous line. Thus, the decision to bolus tPA through the CVC is hypothesized to increase concentration at the site of the thrombus. To date, there have been no randomized controlled trials that compare central to peripherally dosed thrombolytics. To our knowledge, there has been one published case report that describes CVC-directed thrombolytics, which also had a favorable outcome.^5^ Overall, the mortality from massive PE is approximately 15%, but increases to almost 65% when the patient has a cardiac arrest.^6^ In our case, immediate surgical or endovascular therapy was not feasible due to the patient’s instability and the CVC-directed dose of thrombolytics in conjunction with cardiopulmonary resuscitation was associated with her survival.

## CONCLUSION

Patients in cardiac arrest or peri-arrest in the setting of PE may benefit significantly from a bolus of tissue plasminogen activator through a central venous catheter. In the future, more evidence is needed to analyze CVC-directed tPA and its effect on morbidity and mortality.
